# Quantification of the Bioactivity of Ethanolic Extract From Phoenix dactylifera

**DOI:** 10.7759/cureus.56391

**Published:** 2024-03-18

**Authors:** Jeesha Soni, Suganya Panneer Selvam, Rajeshkumar Shanmugam, Ramya Ramadoss, Sandhya Sundar

**Affiliations:** 1 Department of Oral Biology, Saveetha Dental College and Hospitals, Saveetha Institute of Medical and Technical Sciences, Chennai, IND; 2 Nanobiomedicine Lab, Centre for Global Health Research, Saveetha Medical College and Hospital, Saveetha Institute of Medical and Technical Sciences, Chennai, IND

**Keywords:** phoenix dactylifera, polyphenols, ethanol, date seeds, anti-oxidant, anti-inflammatory

## Abstract

Aim: This study aims to quantitatively assess the anti-inflammatory and antioxidant activities of the ethanolic extract of *Phoenix dactylifera* seeds.

Materials and methods: Around 30 seeds of *Phoenix dactylifera* were collected, crushed, and powdered; 10 gm of powder was added to 100 ml of ethanolic extract and boiled for further analysis. Egg albumin denaturation assay and hydroxyl radical scavenging assay were done to evaluate the anti-inflammatory and antioxidant activity, respectively. An independent t-test was used to compare the anti-inflammatory and antioxidant potential of the ethanolic extract of *Phoenix dactylifera *using SPSS Statistics version 22.0 (IBM Corp. Released 2013. IBM SPSS Statistics for Windows, Version 22.0; Armonk, NY: IBM Corp.), and values less than 0.05 are considered statistically significant.

Results: The seeds of *Phoenix dactylifera* have potent anti-inflammatory and antioxidant properties. Both anti-inflammatory and antioxidant properties improved with higher concentrations and were comparable to the control substances diclofenac sodium, vitamin E, and ascorbic acid, respectively. The most significant anti-inflammatory and antioxidant effect was observed at a dosage of 50 μL, with a p-value of 0.001.

Conclusion: To conclude, we found that the ethanolic extract of *Phoenix dactylifera* has anti-inflammatory and antioxidant activity, which can further be used for the improvement of pharmaceuticals.

## Introduction

Date seeds, scientifically known as *Phoenix dactylifera* (*P. dactylifera*), are highly regarded for their numerous advantages and have long been an integral part of the diet. It is evident that date seeds possess significant nutritional value and, despite their high sugar content, contribute to a low glycemic index diet, thereby aiding in the prevention of chronic illnesses [1]. Furthermore, documented research highlights several benefits associated with date seeds, including their hepatoprotective, nephroprotective, anticancer, and antimicrobial properties. These seeds are rich in essential nutrients, serve as an excellent source of dietary fibre, and are hence utilised in various food-related, medicinal, and cosmetic applications [2].

In recent decades, inflammation has emerged as a significant risk factor for a wide range of human disorders. Acute inflammation is a transient and self-contained response that the body's defence mechanisms efficiently manage to restore equilibrium. On the other hand, persistent inflammatory reactions characterised by inflammatory cell infiltration, barrier loss, and excessive cytokine production contribute to the development of chronic disorders. Reducing chronic inflammation has proven effective in the treatment of numerous human conditions [3]. Inflammation plays a significant role in prevalent conditions such as rheumatoid arthritis, atherosclerosis, and asthma. It is the body's physical reaction against harmful substances, triggered by physical trauma, chemical agents, heat, immune responses, or microbial influence [4,5]. Inflammation, despite being a defence mechanism, can initiate, sustain, or exacerbate the underlying disease due to the numerous mechanisms and mediators involved. Although research on inflammatory disorders is ongoing, the adverse effects associated with currently available anti-inflammatory drugs pose a considerable challenge when used for therapeutic purposes. Consequently, there is a need to develop novel anti-inflammatory drugs that are both potent and have fewer adverse effects.

Antioxidants play a crucial role in many nutritional supplements, nutraceuticals, and functional food additives by exerting their health-enhancing effects through the inhibition of oxidation processes. The activity of antioxidants can be evaluated using various assays, including HAT, ET, reducing power, and metal chelation [6,7]. Date seed oil contains saturated and unsaturated fatty acids, as well as phenolic compounds and tocopherols such as oleic and lauric acid. These components make date seed oil suitable for the production of cosmetics, food formulations, and medicines [8]. Nanotechnology is a specialty associated with material science and biology, and many natural product extracts have been discovered to have a variety of pharmacological and antioxidant results [9-11]. The previous studies were mostly concentrated on the infusion of nanoparticles with the extract [12]. We aim to prepare a simple ethanolic extract from the seeds of *P. dactylifera*. Also, most of the mouthwashes recommended by health professionals contain alcohol and various other chemicals, such as triclosan, that cause various side effects ranging from taste disturbance to allergic contact stomatitis. The objective of this research was to analyse the anti-inflammatory and antioxidant qualities of the ethanolic extract of *P. dactylifera* seeds and explore their potential use in enhancing antibiotics and developing a mouthwash to combat oral pathogens while promoting oral hygiene.

## Materials and methods

Preparation of the extract

**Figure 1:**
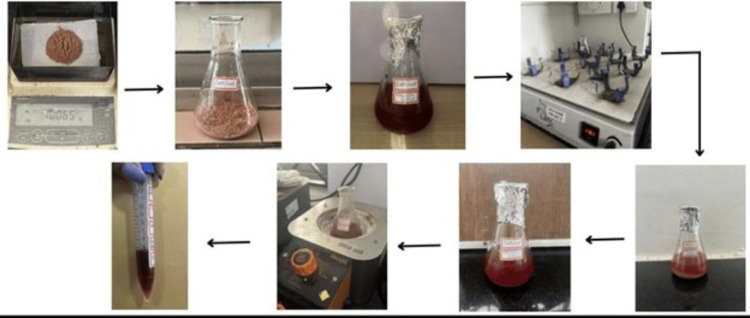
Preparation of the extract from the seeds of P. dactylifera After weighing the 10 gm of extract and transferring it to the beaker of 100 ml ethanol, the solution was mixed using an orbital shaker. The extract was boiled to reduce it to 5 ml (as per the arrows). *P. dactylifera*: *Phoenix dactylifera*

The seeds of *P. dactylifera* were extracted and finely ground, resulting in a fine powder. A beaker containing 10 g of the powder and 100 ml of ethanoic acid was used for the mixture. To ensure uniform mixing, the beaker was placed in an orbital shaker for a few days. Subsequently, the extract was boiled for 5 to 10 minutes to reduce its volume to 5 ml (Figure 1). The filtered extract was transferred to a tube and centrifuged before being used for further analysis.

Anti-inflammatory activity

Albumin Denaturation Assay

Pursuant to the modified methodology indicated by Muzushima and Kabayashi, the albumin denaturation test was carried out to investigate *P. dactylifera*'s anti-inflammatory properties. A tiny amount of 1N hydrochloric acid was used to bring the pH of the amalgamation down to 6.3 after varying quantities of *P. dactylifera* extract (10, 20, 30, 40, and 50 ml) were combined with 0.05 ml of bovine serum albumin (1% aqueous solution). The samples were heated to 55°C in a water bath for 30 minutes, following 20 minutes of incubation at room temperature. After cooling, spectrophotometry was used to determine the absorbance at 660 nm. The percentage of protein denaturation that occurred was estimated using the following equation, using diclofenac sodium as the reference standard:

% inhibition = Absorbance control - Absorbance of sample x 100/ Absorbance of control

Egg Albumin Denaturation Assay

A 5 ml solution was generated for the egg albumin denaturation of the test by diluting 0.2 ml of hen's egg albumin extraction mixed with 2.8 ml of freshly prepared pH 6.3 phosphate-buffered saline. *P. dactylifera* preparations (10 μL, 20 μL, 30 μL, 40 μL, and 50 μL) were added in an assortment of concentrations. The positive control was sodium diclofenac. After that, all of the combinations had been prepared for 15 minutes at 37°C in a water bath. A measurement of the absorbance at 660 nm was conducted after chilling to room temperature.

Antioxidant activity

2,2-Diphenyl-1-picrylhydrazyl (DPPH) ​​​​Method

The antioxidant strength of biogenic zinc oxide nanoparticles was assessed using the DPPH assay. Different amounts of *P. dactylifera* extract (10 μL, 20 μL, 30 μL, 40 μL, and 50 μL) were mixed with 450 μL of 50 mM Tris HCl buffer (pH 7.4) and 1 ml of 0.1 mM DPPH in methanol. The mixture was incubated for 30 minutes, and the decrease in the number of DPPH-free radicals was evaluated based on the absorbance at 517 nm. Ascorbic acid was used as the reference standard, and the percentage of inhibition was calculated using the following equation:

% inhibition= Absorbance of control - Absorbance of test sample x 100/Absorbance of control

Hydroxyl Radical Scavenging Assay

**Figure 2:**
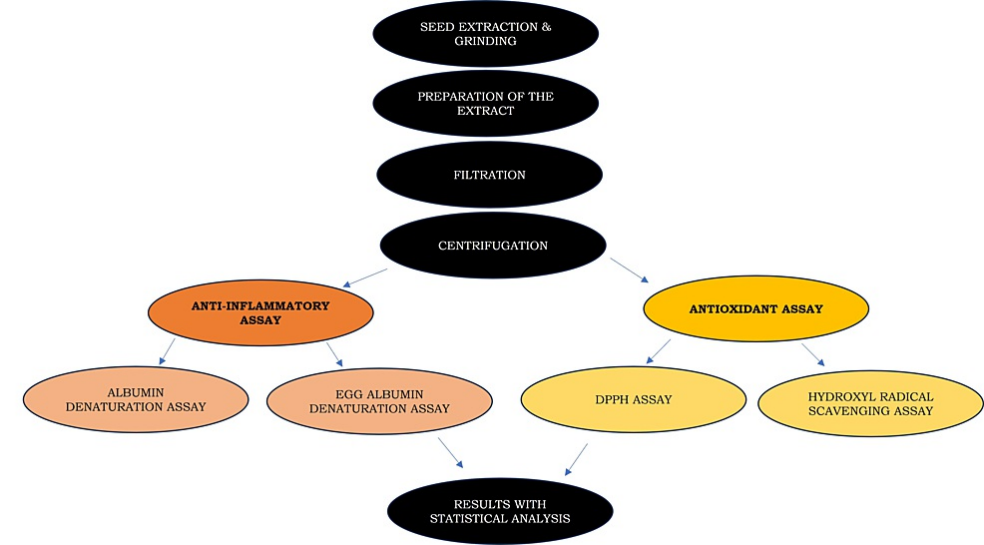
Study design depicted as a flowchart to illustrate the methodological framework

Each fix was prepared from scratch. In a 1.0 mL reaction mixture, solutions of *P. dactylifera* at various concentrations (10 μL, 20 μL, 30 μL, 40 μL, 50 μL) were combined with 100 μL of 28 mM 2-deoxy-2-ribose, 200 μl of 200 μM Fecl3 and 1.04 mM EDTA, and 100 μl of 1.0 (1.0 mM). The extent of deoxyribose degradation at 532 nm was measured against the control solution after one hour of incubation at 37°C. Vitamin E was used as the positive control. The values were tabulated in Microsoft Excel (Microsoft Corporation, Redmond, WA, USA) and transferred to SPSS Statistics version 22.0 (IBM Corp. Released 2013. IBM SPSS Statistics for Windows, Version 22.0. Armonk, NY: IBM Corp.) for statistical analysis. The study's overall framework has been articulated in Figure 2.

## Results

Anti-inflammatory activity

In this study, the impact of *P. dactylifera* seed ethanolic extract on a measure related to concentration was examined across different concentration levels, ranging from 10 to 50 units. The mixtures containing varying concentrations (10 μL, 20 μL, 30 μL, 40 μL, 50 μL) and diclofenac sodium were observed. With a sample size of 3 for both the *P. dactylifera* seeds ethanolic extract and the standard groups at each concentration level, a statistically significant difference (p=0.001) was observed consistently (Tables 1-2). The anti-inflammatory activity of *P. dactylifera* seed extract was significant, with a percentage of inhibition comparable to the standard diclofenac sodium, indicating substantial anti-inflammatory properties of *P. dactylifera*. This suggests a potential influence of the extract on the measured parameter, prompting further exploration into its effects and potential applications in relation to concentration-related factors.

**Table 1:** The anti-inflammatory activity of P. dactylifera extract was assessed using a BSA assay at various concentrations using an independent t-test, and the values are statistically significant (p<0.05) The concentration of the extract was taken in μL. The standard used was diclofenac sodium. The standard deviation shares the same units as the mean, which in turn are identical to the units of the associated random variable (percentage of inhibition). *P. dactylifera*: *Phoenix dactylifera, *BSA: bovine serum albumin

Concentration	Group	N	Mean	Std. deviation	p-value
10	*P. dactylifera* seeds ethanolic extract	3	39.0000	1.00000	0.001
	Standard	3	47.0000	1.00000	
20	*P. dactylifera* seeds ethanolic extract	3	52.0000	1.00000	0.001
	Standard	3	60.0000	1.00000	
30	*P. dactylifera* seeds ethanolic extract	3	64.0000	1.00000	0.001
	Standard	3	72.0000	1.00000	
40	*P. dactylifera* seeds ethanolic extract	3	70.0000	1.00000	0.001
	Standard	3	78.0000	1.00000	
50	*P. dactylifera* seeds ethanolic extract	3	76.0000	1.00000	0.001
	Standard	3	84.0000	1.00000	

**Table 2:** The anti-inflammatory activity of P. dactylifera extract was measured using an EA assay at various concentrations using an independent t-test, and the values are statistically significant (p<0.05) The concentration of the extract was taken in μL. The standard used was diclofenac sodium. The standard deviation shares the same units as the mean, which in turn are identical to the units of the associated random variable (percentage of inhibition). *P. dactylifera*: *Phoenix dactylifera*

Concentration	Group	N	Mean	Std. deviation	p-value
10	*P. dactylifera* seeds ethanolic extract	3	52.0000	1.00000	0.001
	Standard	3	55.0000	1.00000	
20	*P. dactylifera* seeds ethanolic extract	3	60.0000	1.00000	0.001
	Standard	3	64.0000	1.00000	
30	*P. dactylifera* seeds ethanolic extract	3	63.0000	1.00000	0.001
	Standard	3	69.0000	1.00000	
40	*P. dactylifera* seeds ethanolic extract	3	67.0000	1.00000	0.001
	Standard	3	72.0000	1.00000	
50	*P. dactylifera* seeds ethanolic extract	3	74.0000	1.00000	0.001
	Standard	3	81.0000	1.00000	

Antioxidant activity

In our study, the results of an experiment involving DPPH and H2O2 assays were tabulated at various concentrations or time points (10 μL, 20 μL, 30 μL, 40 μL, 50 μL) with the reference standards vitamin E and ascorbic acid. Each concentration/time point was assessed with both a standard and an extract, possibly indicating different treatments or samples. The mean values for the standard and extract measurements show differences in the absorbance or response at specific wavelengths (517 nm for DPPH and 532 nm for H2O2). Generally, as the concentration or time point increases, there is an observable trend in the standard and extract measurements, suggesting varying degrees of impact on the absorbance or response of both DPPH and H2O2 assays. The antioxidant property of *P. dactylifera*, as well as its percentage of inhibition, was found to be comparable to the standard values of ascorbic acid and vitamin E, indicating confirmed antioxidant qualities of *P. dactylifera*. Statistical analysis, as indicated by p-values, highlights significant differences in absorbance or response between the standard and extract at certain concentrations/time points, emphasising the potential efficacy or influence of the extracts compared to the standard treatments in these assays (Table 3).

**Table 3:** The antioxidant activity of P. dactylifera extract was determined using DPPH and H2O2 assays at various concentrations with an independent t-test, and the values are statistically significant (p<0.05) The concentration of the extract was taken in μL. The standard used was ascorbic acid and vitamin E for the DPPH and H2O2 assays, respectively. The standard deviation shares the same units as the mean, which in turn are identical to the units of the associated random variable (percentage of inhibition). DPPH: 2,2-diphenyl-1-picrylhydrazyl, H2O2: hydrogen peroxide

Assay	Concentration	Group	N	Mean	Std. deviation	p-value
DPPH (517 nm)	10	standard	3.00	66.33	0.86	0.21
		extract	3.00	65.24	0.95	
	20	standard	3.00	78.52	1.01	0.01
		extract	3.00	74.36	1.01	
	30	standard	3.00	85.78	0.43	0.01
		extract	3.00	83.38	0.81	
	40	standard	3.00	88.51	0.31	0.00
		extract	3.00	86.52	0.36	
	50	standard	3.00	93.15	0.00	0.00
		extract	3.00	92.21	0.23	
H2O2 (532 nm)	10	standard	3.00	51.34	0.28	0.00
		extract	3.00	50.21	0.05	
	20	standard	3.00	56.75	0.26	0.00
		extract	3.00	52.80	0.31	
	30	standard	3.00	66.22	0.20	0.02
		extract	3.00	65.49	0.29	
	40	standard	3.00	78.47	0.33	
		extract	3.00	76.24	0.19	0.00
	50	standard	3.00	89.27	0.74	
		extract	3.00	82.94	0.69	0.00

## Discussion

In our study, *P. dactylifera* ethanolic extract was observed to have good anti-inflammatory and antioxidant activity. Fruits contain numerous antioxidant compounds, and accurately determining the antioxidant capacity of a specific chemical is challenging [11]. Various methods have been used to measure the antioxidant potential of different plant materials, often assessing their ability to scavenge specific radicals. The findings of this study demonstrate that the aqueous ethanolic extract significantly reduced the production of free radicals, as determined by the DPPH test. The strong antioxidant activity may be attributed to the high content of phytochemical components in the aqueous ethanolic extract, which exhibited superior free radical scavenging compared to the aqueous date extract. Previous studies also concluded Iranian dates as an antioxidant-functional food after the FRAP test, as they have the highest antioxidant potential [12]. In another study, *P. dactylifera* in an ethanolic extract that was aqueous was considerably similar to our results, which unravelled the antioxidant, anti-inflammatory, and anti-tumoral properties of the dates seeds [13].

In a previous study, it was mentioned that *P. dactylifera* fruit, by using the DPPH assay method using liver enzymatic systems, evidently proved the antioxidant activity, which coherently lines up with our study where we used seed [14]. The anti-inflammatory activity increases with increased concentration. The presence of compounds such as quercetin, rutin, caffeic acids, and gallic acids in the dates is responsible for their anti-inflammatory activities [15]. Hence, it aids in the future development of anti-inflammatory drugs or can be added as an adjuvant to any anti-inflammatory drug.

The aqueous ethanolic extract of *P. dactylifera* contained bioactive elements such as quercetin, known for its anti-inflammatory properties. The anti-inflammatory activity of *P. dactylifera* can also be attributed to its antioxidant activity. The *P. dactylifera* extract may have strong anti-inflammatory effects because it has many active ingredients that work together to stop the production, release, and activity of prostaglandins. The rate of inflammation gradually decreased over time as the immune system naturally degraded it, according to the results of several groups treated with ethanol. *P. dactylifera* has antioxidant qualities and contains a variety of active compounds, including alkaloids, tannins, flavonoids, terpenes, and sugars [16,17]. Date palm seeds were found to have superior antifungal properties when compared with Saw palmetto seeds, whereas, in our study, we identified *P. dactylifera* seeds as having potent anti-inflammatory and antioxidant activity [18]. This is because the seeds contain phenolic compounds such as phenolic acids, catechins, tannins, and flavonoids that have the ability to donate hydrogen to DPPH radicals. Recent studies with dates proved that it reduces postpartum haemorrhage after childbirth and overcomes infertility problems; hence, this study can also be extended to analyse the capability of seeds to overcome other common health hazards [19,20].

The ethanolic extract of *P. dactylifera* has demonstrated exceptional anti-inflammatory and antioxidant properties when compared to the standard. However, the present study was limited to locally available date seeds; therefore, a comparative analysis of different date varieties will be conducted in future studies. It is important to note that while the current in vitro assays provide valuable insights, further investigations utilising animal models or clinical trials are imperative to validate the study's findings. Nevertheless, the results obtained are highly significant, as evidenced by the p-value being less than 0.05 in both anti-inflammatory and antioxidant assays. The extract's bioactive compounds render it suitable for incorporation into anti-inflammatory drugs, mouthwash, or oral paste formulations. The study's strength lies in unravelling the medicinal properties of a simple ethanolic extract of date seeds.

## Conclusions

*P. dactylifera* has good antioxidant and anti-inflammatory activity compared to the standard. This pattern hints at a possible inhibitory or modulatory role of the extract in relation to the assays, meriting further investigation into its mechanisms and potential applications in the context of enzyme activity or the specific assay under scrutiny. It can also be used for further improvement of antibiotics and drugs. It can also help in the further improvement of maintaining the oral microbiome by playing a major role in the coherent use of oral pathogens in the making of toothpaste.

## References

[1] Butler AE, Obaid J, Wasif P (2022). Effect of date fruit consumption on the glycemic control of patients with type 2 diabetes: a randomized clinical trial. Nutrients.

[2] Naushad M, Lichtfouse E (2019). Sustainable agriculture reviews 34: date palm for food, medicine and the environment.

[3] Pan MH, Lai CS, Ho CT (2010). Anti-inflammatory activity of natural dietary flavonoids. Food Funct.

[4] Mueller M, Hobiger S, Jungbauer A (2010). Anti-inflammatory activity of extracts from fruits, herbs and spices. Food Chem.

[5] Soni JM, Sardoiwala MN, Choudhury SR, Sharma SS, Karmakar S (2021). Melatonin-loaded chitosan nanoparticles endows nitric oxide synthase 2 mediated anti-inflammatory activity in inflammatory bowel disease model. Mater Sci Eng C Mater Biol Appl.

[6] Al-Dayan N (2021). Biosynthesis of copper oxide nanomaterials using the seeds of date fruits (Phoenix dactylifera L.) and antibacterial activity evaluation. Pak J Biol Sci.

[7] Godugu K, El-Far AH, Al Jaouni S, Mousa SA (2022). Correction: Godugu et al. nanoformulated Ajwa (Phoenix dactylifera) bioactive compounds improve the safety of doxorubicin without compromising its anticancer efficacy in breast cancer. Molecules 2020, 25, 2597. Molecules.

[8] Pandiyan I, Sri SD, Indiran MA, Rathinavelu PK, Prabakar J, Rajeshkumar S (2022). Antioxidant, anti-inflammatory activity of Thymus vulgaris-mediated selenium nanoparticles: An in vitro study. J Conserv Dent.

[9] Vishaka S, Sridevi G, Selvaraj J (2022). An in vitro analysis on the antioxidant and anti-diabetic properties of Kaempferia galanga rhizome using different solvent systems. J Adv Pharm Technol Res.

[10] Prabakar J, Kumaresan S, Kumarr P (2021). In vitro evaluation of cytotoxicity and antioxidant efficacy of bromelain. Int J Dentistry Oral Sci.

[11] Rajeshwaran N, Ramamurthy J, Shanmugam R (2021). Evaluation of antioxidant and anti inflammatory activity of grape seed oil infused with silver nanoparticles an in vitro study. Int J Dentistry Oral Sci.

[12] Mansouri A, Embarek G, Kokkalou E, Kefalas P (2005). Phenolic profile and antioxidant activity of the Algerian ripe date palm fruit (Phoenix dactylifera). Food Chem.

[13] El Abed H, Chakroun M, Fendri I (2017). Extraction optimization and in vitro and in vivo anti-postprandial hyperglycemia effects of inhibitor from Phoenix dactylifera L. parthenocarpic fruit. Biomed Pharmacother.

[14] Kehili HE, Zerizer S, Beladjila KA, Kabouche Z (2016). Anti-inflammatory effect of Algerian date fruit (Phoenix dactylifera). Food Agric Immunol.

[15] Alkhoori MA, Kong AS, Aljaafari MN (2022). Biochemical composition and biological activities of date palm (Phoenix dactylifera L.) seeds: a review. Biomolecules.

[16] Echegaray N, Pateiro M, Gullón B, Misihairabgwi JM, Lorenzo JM (2020). Phoenix dactylifera products in human health - a review. Trends Food Sci Technol.

[17] Alsuhaymi S, Singh U, Al-Younis I (2023). Untargeted metabolomics analysis of four date palm (Phoenix dactylifera L.) cultivars using MS and NMR. Nat Prod Bioprospect.

[18] Barakat AZ, Bassuiny RI, Abdel-Aty AM, Mohamed SA (2020). Diabetic complications and oxidative stress: the role of phenolic-rich extracts of saw palmetto and date palm seeds. J Food Biochem.

[19] Niknami M, Farash M, Rahnavardi M, Maroufizadeh S, Darkhaneh RF (2023). The effect of date fruit consumption on early postpartum hemorrhage: a randomized clinical trial. BMC Womens Health.

[20] Jahromi AR, Mosallanezhad Z, Hosini FS, Jamali S, Sharifi N (2022). The effect of date palm on sexual function in infertile couples: a double-blind controlled clinical trial. BMC Res Notes.

